# Evaluation of the soft tissue biocompatibility of MgCa0.8 and surgical steel 316L in vivo: a comparative study in rabbits

**DOI:** 10.1186/1475-925X-9-63

**Published:** 2010-10-25

**Authors:** Nina Erdmann, Alexandr Bondarenko, Marion Hewicker-Trautwein, Nina Angrisani, Janin Reifenrath, Arne Lucas, Andrea Meyer-Lindenberg

**Affiliations:** 1 Small Animal Clinic, University of Veterinary Medicine Hannover, Bünteweg 9, 30559 Hannover, Germany; 2 Departement of Pathology, University of Veterinary Medicine Hannover, Bünteweg 17, 30559 Hannover, Germany; 3 Institute of Production Engineering and Machine Tools, Leibniz University Hannover, An der Universität 2, 30823 Garbsen, Germany

## Abstract

**Background:**

Recent studies have shown the potential suitability of magnesium alloys as biodegradable implants. The aim of the present study was to compare the soft tissue biocompatibility of MgCa0.8 and commonly used surgical steel *in vivo*.

**Methods:**

A biodegradable magnesium calcium alloy (MgCa0.8) and surgical steel (S316L), as a control, were investigated. Screws of identical geometrical conformation were implanted into the tibiae of 40 rabbits for a postoperative follow up of two, four, six and eight weeks. The tibialis cranialis muscle was in direct vicinity of the screw head and thus embedded in paraffin and histologically and immunohistochemically assessed. Haematoxylin and eosin staining was performed to identify macrophages, giant cells and heterophil granulocytes as well as the extent of tissue fibrosis and necrosis. Mouse anti-CD79α and rat anti-CD3 monoclonal primary antibodies were used for B- and T-lymphocyte detection. Evaluation of all sections was performed by applying a semi-quantitative score.

**Results:**

Clinically, both implant materials were tolerated well. Histology revealed that a layer of fibrous tissue had formed between implant and overlying muscle in MgCa0.8 and S316L, which was demarcated by a layer of synoviocyte-like cells at its interface to the implant. In MgCa0.8 implants cavities were detected within the fibrous tissue, which were surrounded by the same kind of cell type. The thickness of the fibrous layer and the amount of tissue necrosis and cellular infiltrations gradually decreased in S316L. In contrast, a decrease could only be noted in the first weeks of implantation in MgCa0.8, whereas parameters were increasing again at the end of the observation period. B-lymphocytes were found more often in MgCa0.8 indicating humoral immunity and the presence of soluble antigens. Conversely, S316L displayed a higher quantity of T-lymphocytes.

**Conclusions:**

Moderate inflammation was detected in both implant materials and resolved to a minimum during the first weeks indicating comparable biocompatibility for MgCa0.8 and S316L. Thus, the application of MgCa0.8 as biodegradable implant material seems conceivable. Since the inflammatory parameters were re-increasing at the end of the observation period in MgCa0.8 it is important to observe the development of inflammation over a longer time period in addition to the present study.

## Background

To date, stainless steel and titanium are routinely used for internal fracture fixation [1,2]. Besides their high mechanical stability, which is advantageous for rigid fixation of fractured bones, adverse effects such as stress-shielding that result in weakening of the bone and delayed bone healing have been discussed in connection with their use [3]. Further the occurrence of delayed-type hypersensitivity, a cell mediated immune response, has been reported [4]. For these reasons, implant removal is commonly required after fracture healing is completed [5]. To avoid a second surgery and additional inconveniences for the patient as well as unnecessary treatment expenses, it is preferable to choose biodegradable implants. Currently, degradable polymers are available for a variety of non-load bearing applications [6]. However, they have not proven to be successful in osteosynthesis of weight bearing bones [5].

Previous studies have shown the potential suitability of magnesium alloys for an application as biodegradable implants [7-12]. As magnesium alloys have mechanical properties, which are similar to that of cortical bone [13] and a significant higher stability and Young's modulus than polymers [14], they could be promising candidates for osteosynthesis of weight bearing bones.

The development of biodegradable implants demands an appreciation of the cellular and tissue responses that are associated with their implantation and *in vivo* degradation, because they determine the biocompatibility [15]. The response of the host tissue is initiated by the surgical procedure [15] and the presence of the biomaterial itself, which affects the tissue chemically, physically and mechanically [16], and may be divided into three phases [15]. The first phase (so-called early phase) occurs within the first two weeks. It is characterised by initiation, resolution and organisation of the acute and chronic inflammatory host response to the degrading biomaterial [15]. The second phase is dominated by immuno-inflammatory cells, predominantly macrophages, which infiltrate the implantation site. The development of a fibrous capsule is initiated. The process is further enhanced and accompanied by neovascularisation of the capsule during the third phase of the host response [15]. Intensity and duration of the second and third phase reactions are dependent on the degradation rate of the biomaterial [15].

Magnesium alloy implants generally displayed good biocompatibility *in vivo*[17,18]. However, the results of different studies are difficult to compare as the host reactions are not only tissue-, organ- and species-dependent, but also influenced by the size and shape of the implant [15]. The degradation process of a magnesium implant is further influenced by the alloy composition [19,20]. Alloying with calcium is known to enhance the corrosion resistance and mechanical properties of pure magnesium. Binary magnesium calcium alloys with a calcium concentration of 0.6 - 0.8 wt% showed the slowest corrosion rate [21]. With regard to internal fracture fixation, there is necessity to investigate the host reactions of magnesium alloy bone screws at their interface to surrounding tissues. The interface of a screw can be divided into three parts: bone, bone marrow and soft tissue surrounding the screw head, e. g. overlying skeletal muscle. The examination of the bone-implant interface of threaded magnesium calcium alloy pins revealed enhanced osteogenesis and the formation of a fibrous capsule after implantation into rabbit femora [22,23]. Adverse host reactions were not observed [22,23]. The magnesium calcium alloy MgCa0.8 showed good biocompatibility in the bone [23,24]. To the best of the authors' knowledge, detailed studies on the soft tissue biocompatibility of MgCa0.8 are not listed in the current literature.

Therefore, the aim of the present study was to investigate the soft tissue biocompatibility of the biodegradable magnesium calcium alloy MgCa0.8. To quantify the soft tissue biocompatibility of MgCa0.8, host reactions to MgCa0.8 and to commonly used surgical steel, as a control, were investigated *in vivo* at the interface to skeletal muscle.

## Methods

### Implants

**Figure 1:**
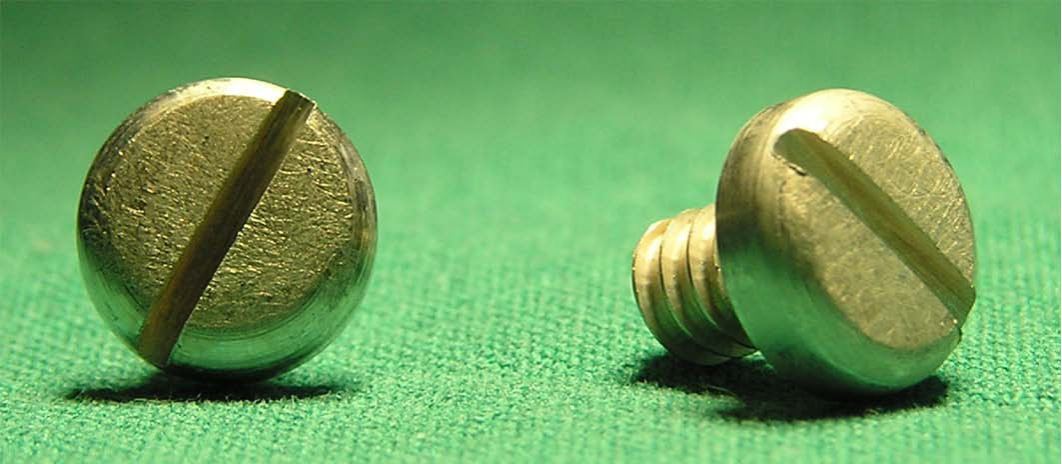
**MgCa0.8 bone screws, which were implanted into rabbit tibiae**. The slotted screw head had diameter of 8.0 mm.

A biodegradable magnesium calcium alloy with a calcium content of 0.8 wt% (MgCa0.8) and commonly used stainless steel 316L (S316L), as a control, were investigated in the present study. Both implant materials were used for the fabrication of cortical bone screws (Figure 1). The MgCa0.8 alloy was produced from pure magnesium (99.8 wt% Mg; Dead Sea Magnesium Ltd, Beer-Sheva, Israel) and the commercially available MgCa30 alloy (30 wt% Ca; Timminco Limited, Toronto, Canada) as previously described [25]. The screws were machined in several steps from extruded bar stocks by turning on a lathe. The feedstock had a diameter of 20 mm for MgCa0.8 and 12 mm for S316L. Separated cylinders were centered and clamped in a CNC-turning center to turn the outer contours of the screw blanks consisting of screw shaft (major diameter: 4.0 mm, length: 6.0 mm) and screw head (head diameter: 8.0 mm). Subsequently, the thread profile (length: 5.0 mm, core diameter: 3.0 mm, pitch (P): 1 mm, thread shape according to ISO 5835) was tapped on the blank in multiple consecutive steps. To keep the mechanical loads applied on the blank minimal during threading, a maximum cutting depth (a_p, max_) of a_p, max _= 0.1 mm for magnesium workpieces and a_p, max _= 0.05 mm for steel bolts was used. Finally the bolts were disconnected and the head geometry was finished by grinding slots with a maximum depth of 1 mm manually.

To remove fabrication process residua the implants were cleaned with acetone and demineralised water. MgCa0.8 was sterilised by exposure to gamma radiation (25 kGy, 6 - 8 h; BBF-Sterilisationsservice GmbH, Kernen, Germany); S316L was sterilised routinely in an autoclave (121°C, 2.3 bar, 60 - 70 min).

### Animal model and study design

The animal experiment was authorized according to the German Animal Welfare Act and registered as number 07/1305. Forty adult, female New Zealand White Rabbits with a mean body weight of 3.81 ± 0.34 kg were randomly assigned to two groups. In the first group, MgCa0.8 screws (n = 48) were implanted into both tibiae of 24 rabbits. In a second group with 16 rabbits, S316L screws (n = 32) were implanted into both tibiae. Surgery was performed under general anaesthesia induced by intramuscular injection of ketamine-hydrochloride (10 mg/kg; Ketamin 10%, CP-Pharma Partner HGmbH, Burgdorf, Germany) and medetomidin (0.125 mg/kg; Domitor^®^, Pfizer Pharma GmbH, Berlin, Germany). After endotracheal intubation anaesthesia was maintained by isoflurane delivered in oxygen (2.5 - 3.5 vol% isoflurane; Isoba^®^, Essex Pharma GmbH, Munich, Germany; oxygen flow: 0.5 - 1.0 l/min). Both hind limbs were clipped and aseptically prepared for surgery. The skin was incised latero-distal of the tibial tuberosity and the cranial tibial muscle was carefully retracted from the tibia. After predrilling with a 3.5 mm burr and tapping the screws were inserted unicortically into the lateral aspect of the tibia slightly proximal of the fibula insertion. In doing so, the screw head was placed underneath the cranial tibial muscle. Finally, the tibial fascia, the subcutis and cutis were closed separately using absorbable suture material (Polysorb^®^, Covidien AG, Dublin, Ireland).

Radiographs were taken immediately after surgery to document correct implant placement. In order to monitor changes at the implantation site, such as gas formation or changes of bone or screw morphology, additional weekly radiographs were taken as a follow up. Physical examinations of both hind limbs were performed every day. Antibiotic and analgesic medication was continued for ten days (enrofloxacin, 10 mg/kg, Baytril^® ^2.5% s. c. once daily, Bayer Animal Health GmbH, Leverkusen, Germany; meloxicam, 0.15 mg/kg s.c. once daily, Metacam^®^, Boehringer Ingelheim Pharma GmbH & Co. KG, Ingelheim am Rhein, Germany). Six animals of MgCa0.8 and four animals of S316L were followed up for two, four, six and eight weeks respectively.

### Histology and immunohistochemistry

**Figure 2:**
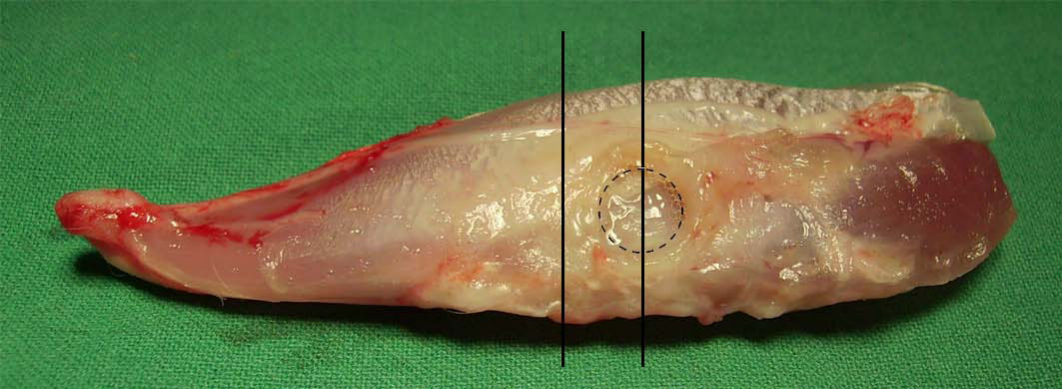
**Explanted cranial tibial muscle of a rabbit after implantation of MgCa0.8 for 6 weeks**. The former position of the screw head is marked by the black circle. To obtain cross sections of the central implantation site for histological and immunohistochemical studies, the muscle was cut into halves first, then one piece of the distal part was embedded in paraffin. The vertical black lines indicate which part of the muscle was used.

At the end of the investigation period, all animals were anesthetised with ketamine-hydrochloride (20 mg/kg; Ketamin 10%, Pharma Partner GmbH, Hamburg, Germany) and xylazine (5 mg/kg; Xylazin 2%, Serumwerk Bernburg AG, Bernburg, Germany) and euthanized by intracardial injection of pentobarbital (230 mg/kg; Narcodorm^®^, CP-Pharma HGmbH, Burgdorf, Germany). The tibialis cranialis muscle was explanted directly after sacrifice. One part of the cranial tibial muscle, which was directly adjacent to the screw head (Figure 2), was fixated in 4% paraformaldehyd for 24 - 48 h to produce cross sections of the periimplant soft tissue interface. After paraffin embedding 2 - 3 μm thick sections were processed using a RM 2255 microtome (Leica Microsystems GmbH, Wetzlar, Germany) and mounted on SuperFrost^® ^Plus slides (Menzel-Gläser, Menzel GmbH & Co KG, Braunschweig, Germany). Three cross sections of each sample were stained with haematoxylin and eosin (H. E.) for histological studies.

An additional group of sections was prepared for immunohistochemical staining. One section of each sample was stained for B-lymphocytes, another for T-lymphocytes. Mouse anti-CD79α (HM 47/A9, Acris Antibodies GmbH, Herford, Germany) and rat anti-CD3 (CD3-12, AbD Serotec, Düsseldorf, Germany) were selected as monoclonal primary antibodies [26]. After deparaffinisation in a descending series of ethanol, endogenous peroxidase was blocked with H_2_O_2 _(0.5% in ethanol) for 30 minutes at room temperature (RT). The sections were then pretreated in boiling sodium citrate buffer (pH 6.0) in the microwave (800 W, 20 min) to demask antigen epitopes. Non-specific binding was reduced by incubation with normal goat serum (1:5) diluted in phosphate-buffered saline (PBS, pH 7.1) for 20 min at RT. The primary antibodies were diluted in PBS containing bovine serum albumin (BSA, 1%). A dilution of 1:800 was used for CD79α, 1:4000 for CD3. Incubation was done overnight in humidified chambers at 4°C. Subsequently, biotinylated secondary antibodies were applied (1:200, 30 min, RT): goat anti-mouse antibodies (Biotinylated Anti-Mouse IgG (H+L), Vector Labs, Burlingame, CA) for CD79α and goat anti-rat antibodies (Biotinylated Anti-Rat IgG (H+L), Vector Labs, Burlingame, CA) for CD3. Following, the avidin-biotin-peroxidase complex system (Vectastain^® ^Elite ABC Kit, Vector Labs, Burlingame, CA) was added. After an incubation of 30 min at RT peroxidase activity was visualised using 3,3-diaminobenzidine-tetrahydrochloride (DAB, 0.05%) and H_2_O_2 _(0.03%) in PBS. Counterstaining was performed with haemalum (20 sec). Finally, the sections were rehydrated in an ascending series of ethanol and cover-slipped with Roti^®^-Histokit II (Carl Roth GmbH & Co, Karlsruhe, Germany). Positive control sections (rabbit lymph nodes) and negative control sections, in which the antibody was replaced by PBS, were included in all staining runs.

All sections were examined repeatedly with an optical light microscope (Axio Imager Z1, Carl Zeiss MicroImaging GmbH, Germany) to obtain an impression of the most prominent changes. The following parameters were selected for evaluation: fibrous encapsulation, i. e. the grade and thickness of periimplant fibrosis, and the amount of necrosis and tissue cavities occurring within the fibrous tissue. Furthermore, the occurrence of immuno-inflammatory cells was evaluated. Macrophages, giant cells and heterophil granulocytes as well as B- and T-lymphocytes were counted. The final evaluation was carried out three times by one observer applying a semi quantitative score (Table 1). The mean score value was calculated for each sample.

**Table 1:** Scoring system for the histological and immunohistochemical evaluation.

Evaluated parameter		Grade	Score
(A)	Fibrosis	absent	0
		minor	1
		mild	2
		moderate	3
		severe	4

(B)	Tissue cavities	absent	0
		minor	1
		mild	2
		moderate	3
		severe	4

(C)	Necrosis	absent	0
		minor	1
		mild	2
		moderate	3
		severe	4

(D)	Cellular infiltrations:	Cells of each type per field of view:	Score
	• Macrophages	0	0
	• Giant cells	1 to 5	1
	• Heterophil granulocytes	6 to 10	2
	• B-lymphocytes	11 to 15	3
	• T-lymphocytes	16 to 20	4
		>20	5

To determine (A) the grade of fibrosis, i. e. thickness and dimension of the periimplant fibrous capsule, and (B) the amount of tissue cavities within the fibrous tissue, the sections were analysed at a magnification of 50× (eyepiece 5×, lens 10×). A magnification of 400× (eyepiece 40×, lens 10×) was used to evaluate (C) the amount of necrosis and (D) the amount of different cellular infiltrations. Therefore, ten randomly chosen microscopic fields of view, that were located within the periimplant fibrous tissue, were analysed and the mean score value was calculated for each section.

### Statistical analysis

Statistical analysis was performed with SPSS 17.0 software package (SPSS Inc., Chicago, USA). Non parametrical tests (Mann-Whitney-Tests) were calculated to determine differences between the two implant materials for each evaluated parameter at each time point. The level of significance was defined as p < 0.05.

## Results

### Clinical and radiological outcome

**Figure 3:**
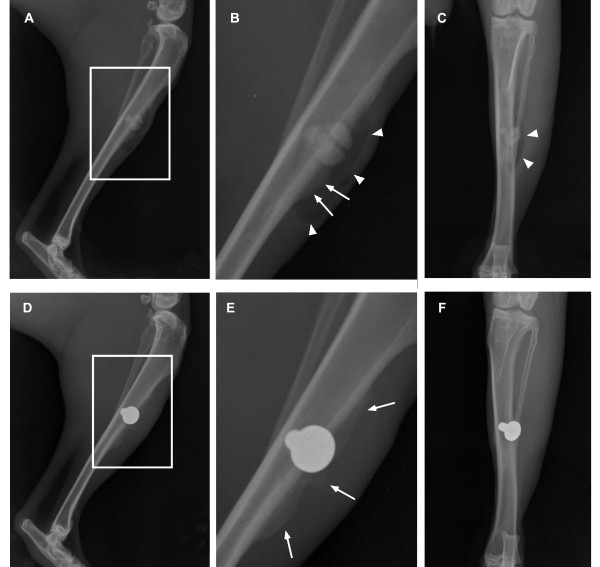
**Radiographs of two rabbit tibiae four weeks post operation**. (A) Medio-lateral projection of a rabbit tibia with an implanted MgCa0.8 screw. (B) Magnification corresponding to the white rectangle in (A). (C) Cranio-caudal projection of the same tibia. New bone (white arrows) and accumulation of gas (white triangles) are clearly observable at the implantation site of MgCa0.8. (D) Medio-lateral projection of a rabbit tibia with an implanted S316L screw. (E) Magnification corresponding to the white rectangle in (D). (E) Cranio-caudal projection of the same tibia. At the implantation site of S316L only new bone (white arrows) is detectable.

Clinically, both implant materials were well tolerated. All animals showed mild wound swelling and reddening resulting from postoperative haematoma, which completely resolved not later than 14 days after surgery. Neither infections nor suture intolerance occurred during the postoperative follow up. Mild unilateral hind limb lameness was found in three cases and cured after treatment with meloxicam (0.15 mg/kg; Metacam^®^, Boehringer Ingelheim Pharma GmbH & Co. KG, Ingelheim am Rhein, Germany) administered for four to five days. New bone development could be radiographically detected near the screw head in all animals of both material groups (Figure 3), which increased in size as well as in radiographic density throughout the follow up. Clinically and radiographically, periimplant emphysema was detected in MgCa0.8 only (Figure 3). One week postoperatively the radiographic examination revealed a radiolucent gas margin surrounding the screw head of all MgCa0.8 implants except for three. In these three cases periimplant gas was detected for the first time after two, four and five weeks respectively. Throughout the follow up, gas shadows increased in size as periimplant gas accumulated to form a bubble around the screw head. In two cases periimplant gas bubbles dissolved without a special treatment five and six weeks postoperatively. In all other cases periimplant gas bubbles were noted throughout the remaining follow up.

The dissection of the cranial tibial muscle showed that MgCa0.8 and S316L provoked a similar tissue response. In both implant materials, a layer of soft tissue had formed around the screw head, which was adherent to the overlying muscle. Strong adhesion of the periimplant soft tissue or the cranial tibial muscle tissue to the surface of the screw head was not observed.

### Histological evaluation

**Figure 4:**
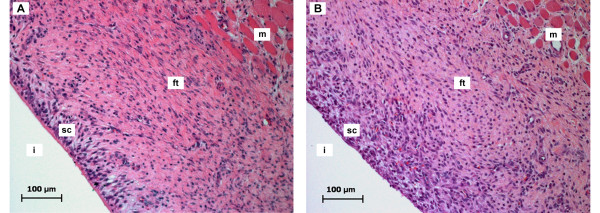
**Image sections of cranial tibial muscle cross sections two weeks post operatively (H. E., 200×)**. (A) MgCa0.8 implant site and (B) S316L implant site, respectively. Note the fibrous tissue (ft) that formed between the cranial tibial muscle (m) and the implant site (i) and is surrounded by a layer of synoviocyte-like cells (sc) at its interface to the implant in both implant materials.

**Figure 5:**
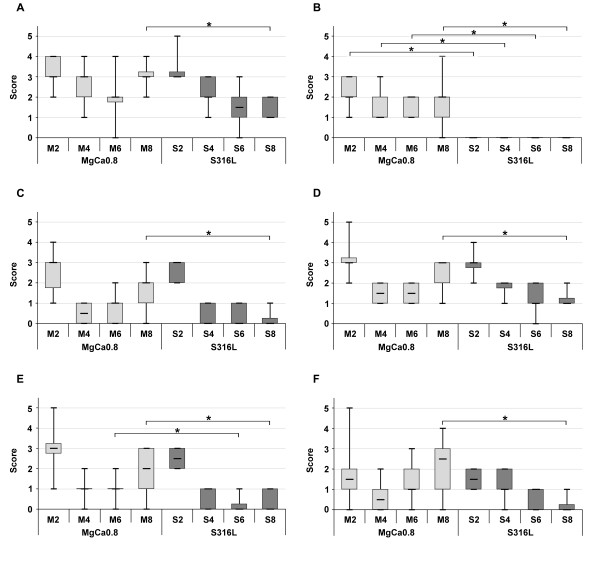
**Boxplots of the histological evaluation of cranial tibial muscle cross sections after implantation of MgCa0.8 (light grey boxplots) and S316L (dark grey boxplots) for two, four, six and eight weeks (two weeks is represented as M2 for MgCa0.8 and as S2 for S316L, four weeks as M4 for MgCa0.8 and S4 for S316L, etc.)**. The boxplots are representing the amount of (A) periimplant fibrosis, (B) tissue cavities, (C) necrosis, (D) macrophages, (E) giant cells and (F) heterophil granulocytes. Non parametrical tests (Mann-Whitney-Tests) were calculated to determine differences between the implant materials. Stars represent significant difference, which was defined as p < 0.05.

A layer of fibrous tissue was noted in histological specimens of MgCa0.8 (Figure 4A) and S316L (Figure 4B) that had formed between the screw head and the overlying muscle. It was demarcated by a single layer of synoviocyte-like cells, predominantly cubic or columnar cells with acidophilic cytoplasm, at its implant interface. The surface of the cells consisted of acellular, fibrinoid-like eosinophilic material consistent with apocrinic secretion. The thickness of the fibrous layer changed throughout the follow up. Two weeks after surgery a moderately developed fibrous tissue layer was detected in MgCa0.8 and S316L (median score: 3.0; p = 0.79) (Figure 5A). The thickness gradually decreased in S316L (median score: 1.0 after eight weeks). On the contrary, a gradual decrease was only noted within the first six weeks in MgCa0.8 (median score: 3.0 after four weeks, median score: 2.0 after six weeks). After eight weeks the amount of fibrous tissue was increasing again (median score: 3.0). A significant difference between MgCa0.8 and S316L could only be noted after eight weeks (p < 0.001).

**Figure 6:**
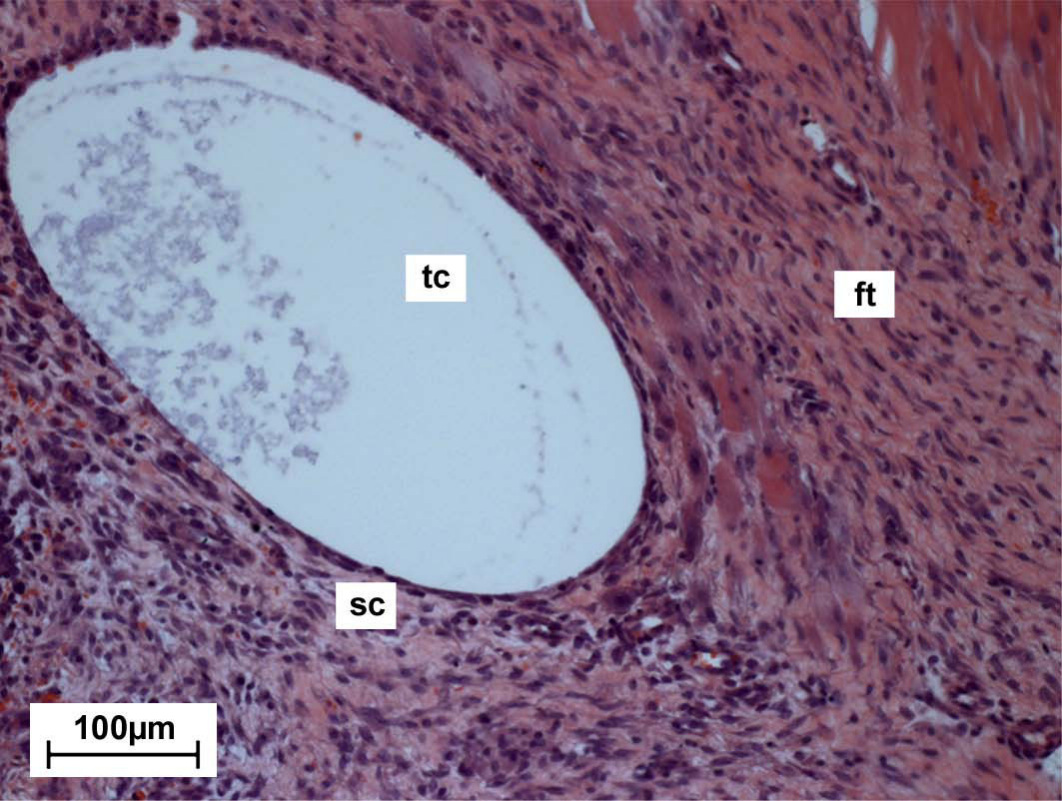
**Image section of an MgCa0.8 implant site two weeks postoperatively (H. E., 200×)**. Within the periimplant fibrous tissue (ft) a tissue cavity (tc) is clearly observable. The tissue cavity is surrounded by a synoviocyte-like cell layer (sc).

Cavities were solely detected within the fibrous tissue of MgCa0.8 implants and were lined with the same kind of synoviocyte-like cell layer detected at the fibrous tissue implant interface (Figure 6). Compared to the entire fibrous layer the tissue around the cavities did not show any changes of its morphology. Throughout the follow up the size and quantity of the tissue cavities slightly varied decreasing from week two (median score: 2.0) to week four (median score: 1.0) and re-increasing at the end of the implantation period (median score: 2.0 after six and eight weeks) (Figure 5B).

**Figure 7:**
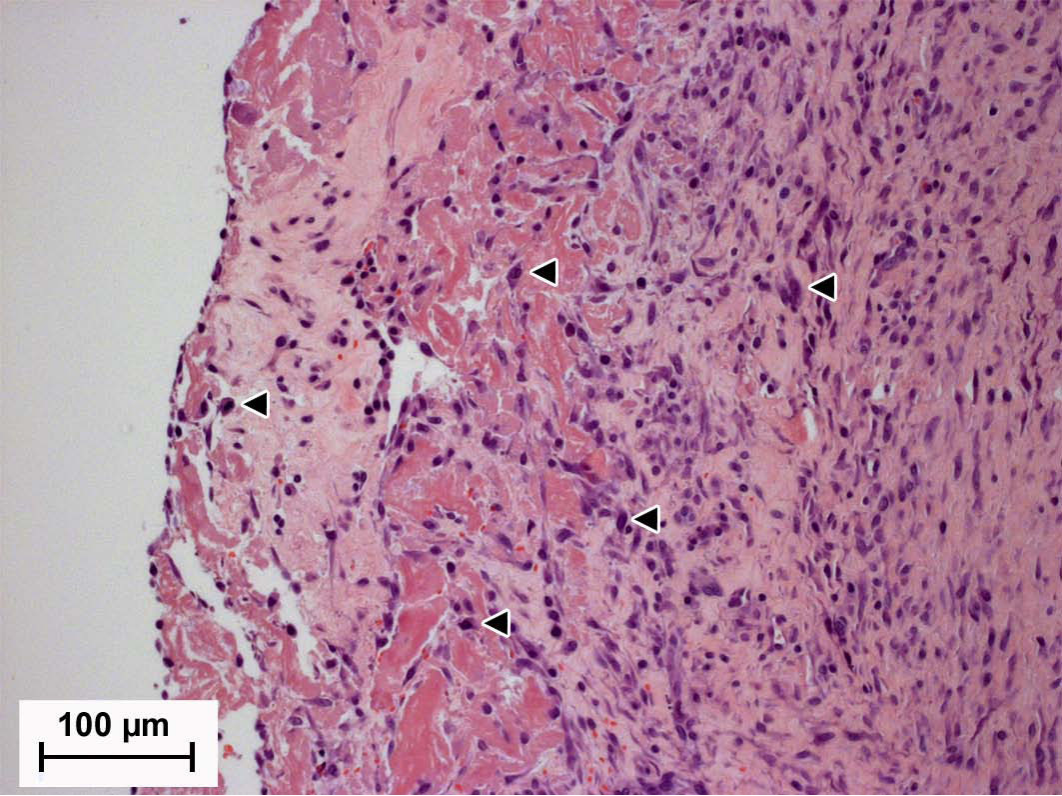
**Image section of an S316L implant site two weeks postoperatively showing moderate necrosis and mild infiltration with macrophages within a moderately developed fibrous layer (H. E., 200×)**. The area of necrosis at the edge of the fibrous tissue is clearly observable. Macrophages are exemplarily represented by triangles.

**Figure 8:**
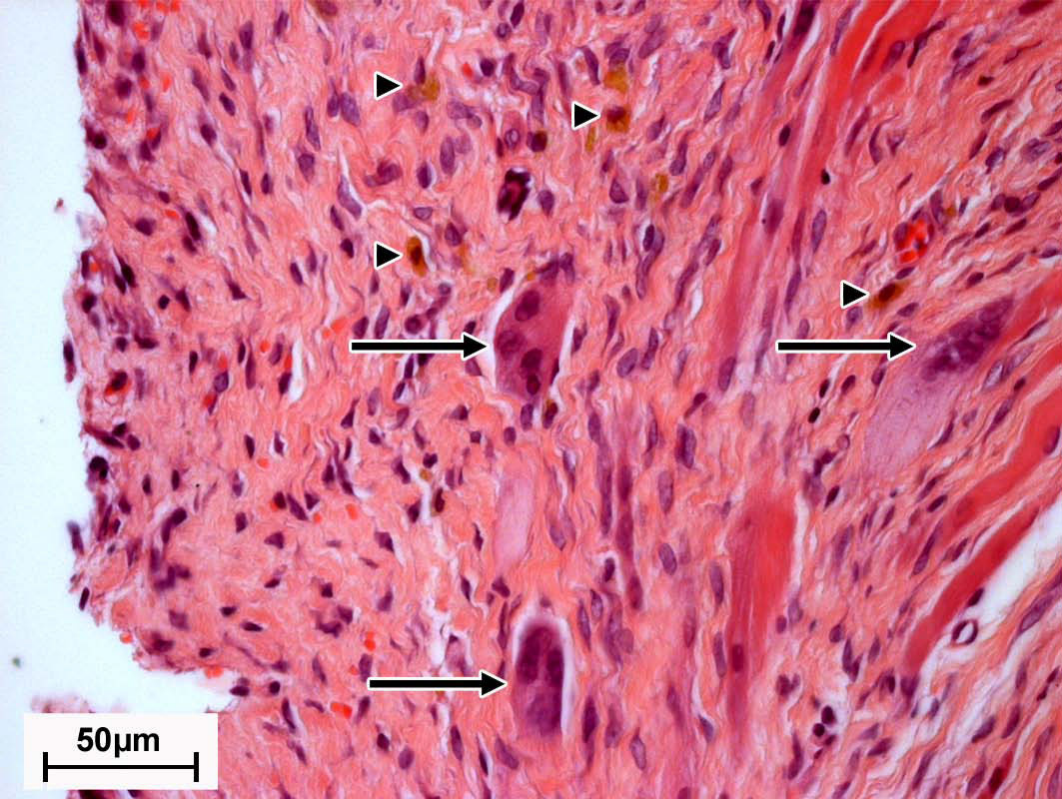
**Image section of the periimplant fibrous tissue of an MgCa0.8 screw four weeks postoperatively (H. E., 400×)**. Within the fibrous tissue mild infiltrations of giant cells (arrows) and macrophages (triangles) are detectable.

**Figure 9:**
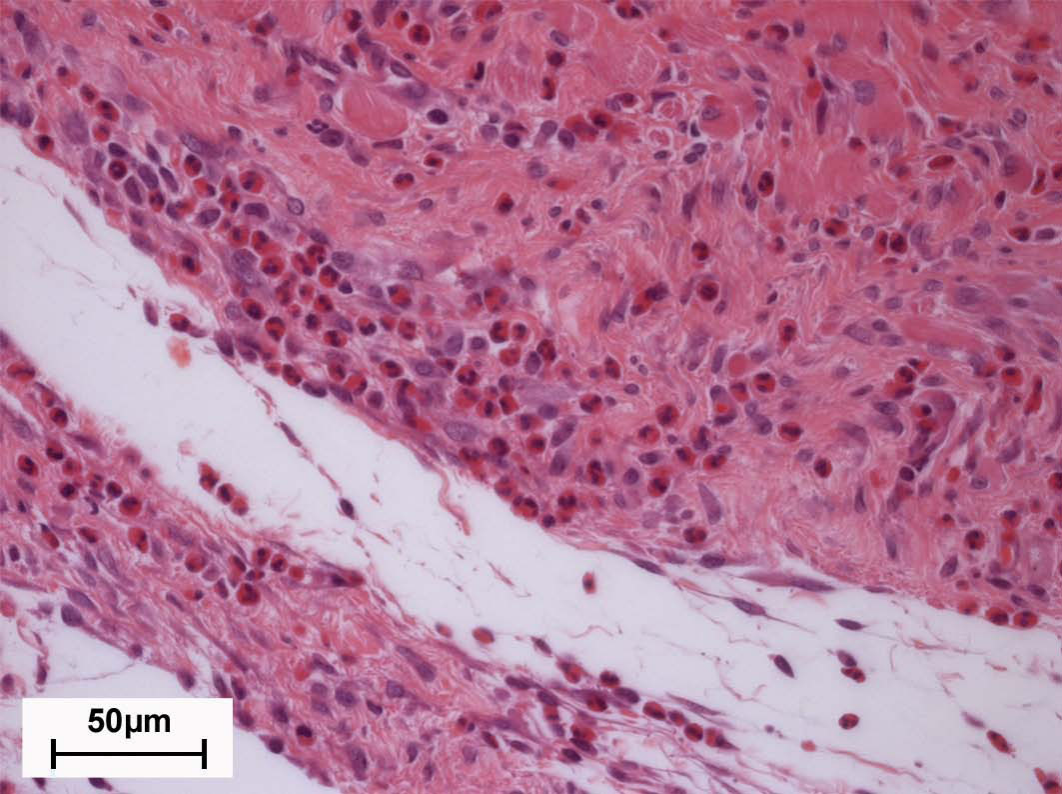
**Image section of the periimplant fibrous tissue of an MgCa0.8 screw eight weeks postoperatively showing moderate infiltrations of heterophil granulocytes that are clearly observable due to their lobulated nucleus and red cytoplasmic granules (H. E., 400×)**.

Furthermore, small spots of necrosis (Figure 7) and infiltrations of immuno-inflammatory cells (Figure 8, Figure 9) were dispersed within the periimplant fibrous tissue of both implant materials. Two weeks after surgery moderate amounts of necrotic tissue were detected in MgCa0.8 and S316L (median score: 3.0; p = 0.91) (Figure 5C). The amount of necrosis was decreasing throughout the follow up in S316L (median score: 0 after four, six and eight weeks). In MgCa0.8, a decrease in necrotic tissue was detected after four weeks (median score: 0.5), but at the end of the observation period the amount of necrosis was increasing again (median score: 1 after six weeks; median score: 2 after eight weeks). Eight weeks after surgery the degree of necrosis was significantly higher in MgCa0.8 than in S316L (p = 0.001).

Furthermore, the quantity of macrophages, giant cells and heterophil granulocytes that occurred within the periimplant fibrous tissue was evaluated (Figure 8, Fig 9). Two weeks after surgery moderate numbers of macrophages (median score: 3.0, MgCa0.8 and S316L) (Figure 5D) and giant cells (MgCa0.8: median score: 3.0; S316L: median score: 2.5) (Figure 5E) were found in all samples. Heterophils were also detected in both groups, but in lower numbers than the other cell types (median score: 1.5; MgCa0.8 and S316L) (Figure 5F). The quantity of cellular infiltrations did not significantly differ between both implant materials two weeks after surgery (macrophages: p = 0.38; giant cells: p = 0.24; heterophils: p = 1.00). Throughout the observation period the number of evaluated cells gradually decreased in S316L (macrophages: median score: 1.0; giant cells and heterophils: median score: 0), whereas in MgCa0.8 a decrease was only noted in the first weeks of implantation. At the end of the observation period the amount of macrophages (median score: 3.0 after eight weeks), giant cells (median score: 2.0 after eight weeks) and heterophils (median score: 2.5 after eight weeks) increased again. A significant difference was determined between the two material groups for week eight after surgery only (macrophages: p = 0.005; giant cells: p = 0.003; heterophils: p = 0.001).

### Immunohistochemical evaluation

**Figure 10:**
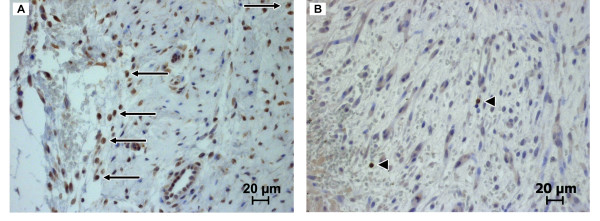
**Image sections of immunohistochemically stained cranial tibial muscle cross sections**. (A) Periimplant fibrous tissue of an MgCa0.8 screw six weeks post operatively (CD79α, 400×). Arrows represent CD79α positive B-lymphocytes. (B) Periimplant fibrous tissue of an S316L screw two weeks postoperatively (CD3, 400×). Triangles indicate CD3 positive T-lymphocytes.

**Figure 11:**
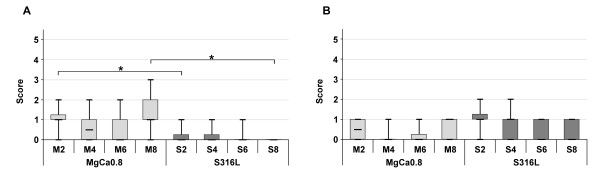
**Boxplots of the immunohistochemical evaluation of cranial tibial muscle cross sections after implantation of MgCa0.8 (light grey boxplots) and S316L (dark grey boxplots) for two, four, six and eight weeks (two weeks is represented as M2 for MgCa0.8 and as S2 for S316L, four weeks as M4 for MgCa0.8 and S4 for S316L, etc.)**. The boxplots represent the amount of (A) B-lymphocytes and (B) T-lymphocytes. Non parametrical tests (Mann-Whitney-Tests) were calculated to determine differences between the implant materials. Stars represent significant difference, which was defined as p < 0.05.

To detect lymphocytes that migrated into the periimplant fibrous tissue, immunohistochemical staining was performed. Immunostaining for CD79α revealed that B-lymphocytes only occurred in low numbers in MgCa0.8 and S316L (Figure 10A, Figure 11A). In MgCa0.8 the amount of B-lymphocytes decreased from week two (median score 1.0) to week four (median score 0.5) and week six (median score 0). Eight weeks postoperatively a re-increase of B-lymphocytes was noted (median score 1). In S316L a median score of 0 was noted for B-lymphocytes two, four, six and eight weeks postoperatively, as B-lymphocytes were only detected in five samples (16%) throughout the follow up. The amount of B-lymphocytes was significantly lower in S316L than in MgCa0.8 two (p = 0.02) and eight weeks (p = 0.001) after surgery. T-lymphocytes also only occurred in low numbers in MgCa0.8 and S316L (Figure 10B, Figure 11B). In MgCa0.8 a median score of 0.5 was noted two weeks postoperatively decreasing to a score of 0 after four and six weeks. Eight weeks after surgery the amount of T-lymphocytes re-increased in MgCa0.8 (median score 1.0). S136L showed a median score of 1.0 after two and four weeks decreasing to a median score of 0 after six and eight weeks. No significant differences between MgCa0.8 and S316L were detected throughout the whole observation period.

The cranial tibial muscle showed physiological morphology in both groups at all time points. Tissue cavities, signs of necrosis and enhanced cellular infiltrates that were seen in MgCa0.8 and S316L implants were restricted to the periimplant fibrous tissue and its transition to the muscle, but none of these alterations was detected within the muscle tissue itself.

## Discussion

The aim of the present study was to assess the soft tissue biocompatibility of MgCa0.8 by quantifying the host reaction at the interface to periimplant skeletal muscle. For this purpose, the severity of the early inflammatory reactions to MgCa0.8 and commonly used S316L, as a control, was compared. Screw shaped implants were inserted into the tibial bone of rabbits. Thus it was possible to keep the screw head in direct contact with the overlying cranial tibial muscle. To obtain enough area of contact between muscle and implant, screws with an exceptionally large screw head were used. The inflammatory host response of the cranial tibial muscle was judged clinically, radiographically and histologically taking the formation of a fibrous capsule, the amount of necrosis and the infiltration with immuno-inflammatory cells such as macrophages, giant cells, heterophil granulocytes and lymphocytes into account. To specify the inflammatory reactions, the occurrence of B- and T-lymphocytes was evaluated by immunohistochemical staining.

Previous *in vivo* studies have shown good biocompatibility of MgCa0.8 in bone [23,24]. Detailed studies on the soft tissue reaction to MgCa0.8 are not described in the current literature. In the present study MgCa0.8 was clinically tolerated comparably well to the control material S316L. Radiographically a radiolucent gas margin was detected around the MgCa0.8 screw head indicating the beginning degradation of MgCa0.8. Hydrogen gas production is well known during the degradation of magnesium [27,28]. The gas either diffuses into periimplant tissues and is eliminated by the blood flow or accumulates in tissue cavities depending on the local gas saturation [29]. It is not yet examined to which extent the production of hydrogen might influence surrounding tissues. Previous *in vivo* studies on other magnesium calcium alloys reported the formation of gas bubbles in the early implantation period, which dissolved after two months without any treatment [22]. In the present study, gas cavities could be detected at the implantation site of MgCa0.8 clinically and radiographically. In contrary to former studies [22] only short implantation times up to eight weeks were investigated and the gas bubbles did not dissolve within the observation period except for two cases. The histological examination of the periimplant soft tissue revealed the presence of tissue cavities as well, which were detected within the fibrous tissue layer of MgCa0.8 and did not form in S316L. Thus they might be associated with the accumulation of hydrogen. In consistence with the clinical results of the present study, the tissue cavities were histologically detected in all time groups of MgCa0.8. Nevertheless, the fibrous tissue around the cavities did not show enhanced cellular infiltrates compared to the entire fibrous layer. Also, no clinically adverse effects were detected due to the gas production. For that we could strengthen the priory reported thesis [22,30] that the formation of hydrogen cavities does not seem to affect the host adversely.

Edwards [31] described that repeated subcutaneous injections of air into the subcutaneous tissue of rats and mice resulted in the development of tissue cavities that were lined by a synovial membrane. The tissue cavities that developed during degradation of MgCa0.8 in the present study were lined by a layer of cells resembling synoviocytes. The interface of the periimplant fibrous tissue to MgCa0.8 and S316L was demarcated by the same kind of synoviocyte-like cell layer. Previous studies described synovial metaplasia, which was defined as a distinct membraneous proliferation of synoviocyte-like cells, at the tissue implant interface as a common response to biomedical implants [32,33]. The mechanisms of synovial metaplasia development remain uncertain, but mechanical factors have been suggested to play an important role [31,33]. It is likely that the development of synovial metaplasia in the present study was the result of repeated mechanical stress of the fibrous tissue by the implant and/or the gaseous accumulations.

The formation of fibrous tissue is a key part of the host response and is well known for conventional osteosynthesis implants [34-36]. Even well integrated implants might be surrounded by an intervening fibrous tissue layer [37]. In the present study a moderately developed fibrous layer formed at the soft tissue interface of MgCa0.8 and S316L. The cranial tibial muscle, which was in direct contact to the screw head, showed a physiological morphology. Within the fibrous layer adjacent to the implants low to moderate amounts of immuno-inflammatory cells and small necrotic spots were diffusely dispersed. To judge the severity of host reactions it is as important to analyse the dimension of the fibrotic layer as well as to evaluate the amount of occurring cells [38]: a highly developed layer with many inflammatory cells is a sign for encapsulation, whereas a moderate extent of fibrosis with few inflammatory cells, as detected in the present study, more likely displays a beginning integration of the implant [39]. Every implantation procedure involves injury of the surrounding tissue and activates inflammatory and wound healing responses [15]. In the normal wound healing process granulocytes occur at the implantation site initially, but at later stages macrophages, which participate in the decontamination of the wound through phagocytosis, are the predominant cells in the reactive tissue adjacent to an implant [40]. In an attempt to phagocytise the implant, which acts as a foreign body, giant cells are formed by the fusion of macrophages [40]. The additional appearance of lymphocytes is a sign for chronic inflammation with humoral and cell mediated immune reactions [40]. In this context, the amount of cells and the time of their occurrence are crucial.

In the present study two major differences were noted for MgCa0.8 and S316L: the course of host reactions and the involvement of lymphocytes. While host reactions started at the same level in both implant materials and proceeded equally during the first six weeks, a contrary course was noted after eight weeks. Two weeks postoperativeley macrophages and giant cells were the predominant cells within the periimplant fibrous tissue. Heterophil granulocytes and lymphocytes only occurred in minor quantities. It is likely that this early inflammatory reaction was induced by the surgical procedure, which always leads to tissue damage and mild inflammation [15]. Resolution of the early inflammatory response and wound healing was concluded, since all examined parameters decreased during the first six weeks. Only the amount of heterophil granulocytes slightly re-increased after six weeks in MgCa0.8. After eight weeks a re-increase of all examined parameters was noted in MgCa0.8, whereas the examined parameters further decreased in S316L. Thus, a significant difference was detected between MgCa0.8 and S316L for all examined parameters eight weeks after surgery except for the amount of T-lymphocytes. It is assumed that the reoccurrence of the inflammatory reactions in MgCa0.8 correlates with the degradation of the implant. Previous *in vivo* studies have shown that the degradation rate of a magnesium implant influences the degree of the inflammatory host response in bone tissue [23]. Gogolewski *et al*. [41] reported that the tissue reaction to degradable polymer implants correlates with the degradation rate as well. Therefore, it is likely that the intensified degradation of MgCa0.8 eight weeks postoperatively induced a restart of the inflammatory host reactions. Nevertheless, the examined parameters did not exceed the moderate level at any time. Thus no severe inflammatory reactions were observed in MgCa0.8. Contrary to that, intense granulomatous inflammatory soft tissue lesions were described with the use of absorbable polymer internal fixation devices [42-44].

In the present study it was further shown that a distinct difference existed between MgCa0.8 and S316L concerning the involvement of lymphocytes, which represent the mediators of humoral and cellular immunity. Humoral immunity reactions were suggested in MgCa0.8, as higher quantities of B-lymphocytes than T-lymphocytes occurred. B-lymphocytes are the origin of humoral immunity. Once they are activated B-lymphocytes differentiate into antibody producing plasma cells [45]. The predominant humoral immune response to MgCa0.8 indicates the presence of soluble antigens such as wear particles of the degrading implant. In contrast, T-lymphocytes occurred in higher quantities than B-lymphocytes at the implantation site of S316L. T-lymphocytes are the mediators of cellular immunity and hypersensitivity reactions. Cell mediated immunity is well known for surgical steel implants, as they contain sensitizers such as nickel and chromium, which can cause hypersensitivity reactions [46]. The involvement of cellular immunity with the use of another magnesium alloy implant has formerly been investigated. In consistence with the results of the present investigations, only minor quantities of T-lymphocytes were detected at the magnesium implant site [18]. Thus cell mediated immunity seems to be of less importance in MgCa0.8 alloy implants.

## Conclusions

Both implant materials were tolerated well since only moderate inflammation, probably induced by the surgical procedure, was detected at the implantation site of MgCa0.8 and S316L. The early inflammatory host response resolved to a minimum during the first six weeks. Main differences between both implant materials were the course of inflammation after eight weeks and the involvement of the immune system. Predominating humoral immunity was observed in MgCa0.8, whereas cell mediated immunity was detected in S316L. However, only low amounts of immuno-inflammatory cells were detected in both groups. Hydrogen cavities that were produced by the degrading implant did not seem to affect the host adversely as they did not influence the extent of the host response. The intensity of the inflammatory host response to both materials did not differ significantly during the first weeks of implantation indicating comparable biocompatibility for MgCa0.8 and S316L. Eight weeks postoperatively inflammation re-increased in MgCa0.8, which might be caused by intensified degradation of MgCa0.8. Nevertheless, the examined parameters did not exceed the moderate level nor indicate rejection of the implant. Thus, the application of MgCa0.8 as biodegradable implant material seems conceivable. It is necessary to investigate the host reaction over a longer time period to observe the further development of inflammation. In addition, coated MgCa0.8 alloys that could reduce the degradation rate and the gas production should be investigated in further studies.

## Competing interests

The authors declare that they have no competing interests.

## Authors' contributions

NE participated in the animal experiment and the histological and immunohistochemical examinations, analysed the data and wrote the manuscript. AB participated in the histological and immunohistochemical examinations. MHT supervised the histological and immunohistochemical examinations. NA participated in the animal experiment and helped to draft the manuscript. JR participated in the animal experiment and the design of the study. AL fabricated and provided the implants. AML initiated and conceived of the study, participated in its design and coordination and participated in the animal experiment. All authors read and approved the final manuscript.

## References

[B1] Pohler OEM Unalloyed titanium for implants in bone surgery Injury 2000 31 Suppl 4 7 13 10.1016/S0020-1383(00)80016-9 11270082

[B2] Disegi JA Eschbach L Stainless steel in bone surgery Injury 2000 31 Suppl 4 2 6 10.1016/S0020-1383(00)80015-7 11270076

[B3] Nagels J Stokdijk M Rozing PM Stress shielding and bone resorption in shoulder arthroplasty J Shoulder Elbow Surg 2003 12 35 39 10.1067/mse.2003.22 12610484

[B4] Hallab N Merritt K Jacobs JJ Metal sensitivity in patients with orthopaedic implants J Bone Joint Surg Am 2001 83-A 428 436 11263649 10.2106/00004623-200103000-00017

[B5] Hofmann GO Biodegradable implants in traumatology: a review on the state-of-the-art Arch Orthop Trauma Surg 1995 114 123 132 10.1007/BF00443385 7619632

[B6] Navarro M Michiardi A Castaño O Planell JA Biomaterials in orthopaedics J R Soc Interface 2008 5 1137 1158 10.1098/rsif.2008.0151 18667387 PMC2706047

[B7] McBride ED Absorbable Metal in Bone Surgery J Am Med Assoc 1938 111 2464 2467

[B8] Verbrugge J Le Matériel Métallique Résorbable En Chirurgie Osseuse La Press Med 1934 23 460 465

[B9] Peuster M Beerbaum P Bach FW Hauser H Are resorbable implants about to become a reality? Cardiol Young 2006 16 107 116 10.1017/S1047951106000011 16553970

[B10] Song G Control of biodegradation of biocompatable magnesium alloys Corros Sci 2007 49 1696 1701 10.1016/j.corsci.2007.01.001

[B11] Zberg B Uggowitzer PJ Löffler JF MgZnCa glasses without clinically observable hydrogen evolution for biodegradable implants Nat Mater 2009 8 887 891 10.1038/nmat2542 19783982

[B12] Zhang E Xu L Yu G Pan F Yang K In vivo evaluation of biodegradable magnesium alloy bone implant in the first 6 months implantation J Biomed Mater Res A 2009 90 882 893 18618719 10.1002/jbm.a.32132

[B13] Staiger MP Pietak AM Huadmai J Dias G Magnesium and its alloys as orthopedic biomaterials: a review Biomaterials 2006 27 1728 1734 10.1016/j.biomaterials.2005.10.003 16246414

[B14] Shikinami Y Okuno M Bioresorbable devices made of forged composites of hydroxyapatite (HA) particles and poly-L-lactide (PLLA): Part I. Basic characteristics Biomaterials 1999 20 859 877 10.1016/S0142-9612(98)00241-5 10226712

[B15] Shive Anderson Biodegradation and biocompatibility of PLA and PLGA microspheres Adv Drug Deliv Rev 1997 28 5 24 10.1016/S0169-409X(97)00048-3 10837562

[B16] Clark AE Hench LL Paschall HA The influence of surface chemistry on implant interface histology: a theoretical basis for implant materials selection J Biomed Mater Res 1976 10 161 174 10.1002/jbm.820100202 1254612

[B17] Hänzi AC Gunde P Schinhammer M Uggowitzer PJ On the biodegradation performance of an Mg-Y-RE alloy with various surface conditions in simulated body fluid Acta Biomater 2009 5 162 171 10.1016/j.actbio.2008.07.034 18762463

[B18] Witte F Ulrich H Rudert M Willbold E Biodegradable magnesium scaffolds: Part 1: appropriate inflammatory response J Biomed Mater Res A 2007 81 748 756 17390368 10.1002/jbm.a.31170

[B19] Witte F Kaese V Haferkamp H Switzer E Meyer-Lindenberg A Wirth CJ Windhagen H In vivo corrosion of four magnesium alloys and the associated bone response Biomaterials 2005 26 3557 3563 10.1016/j.biomaterials.2004.09.049 15621246

[B20] Krause A von der Höh N Bormann D Krause C Bach F Windhagen H Meyer-Lindenberg A Degradation behaviour and mechanical properties of magnesium implants in rabbit tibiae J Mater Sci 2010 45 624 632 10.1007/s10853-009-3936-3

[B21] Drynda A Hassel T Hoehn R Perz A Bach F Peuster M Development and biocompatibility of a novel corrodible fluoride-coated magnesium-calcium alloy with improved degradation kinetics and adequate mechanical properties for cardiovascular applications J Biomed Mater Res A 2010 93 763 775 19653306 10.1002/jbm.a.32582

[B22] Li Z Gu X Lou S Zheng Y The development of binary Mg-Ca alloys for use as biodegradable materials within bone Biomaterials 2008 29 1329 1344 10.1016/j.biomaterials.2007.12.021 18191191

[B23] von der Höh N Rechenberg von B Bormann D Lucas A Meyer-Lindenberg A Influence of different surface machining treatments of resorbable magnesium alloy implants on degradation - EDX-analysis and histology results Mat-wiss u Werkstofftech 2009 40 88 93 10.1002/mawe.200800378

[B24] Thomann M Krause C Bormann D von der Höh N Windhagen H Meyer-Lindenberg A Comparison of the resorbable magnesium alloys LAE442 und MgCa0.8 concerning their mechanical properties, their progress of degradation and the bone-implant-contact after 12 months implantation duration in a rabbit model Mat-wiss u Werkstofftech 2009 40 82 87 10.1002/mawe.200800412

[B25] von der Höh N Bormann D Lucas A Denkena B Hackenbroich C Meyer-Lindenberg A Influence of Different Surface Machining Treatments of Magnesium-based Resorbable Implants on the Degradation Behavior in Rabbits Adv Eng Mater 2009 11 B47 B54 10.1002/adem.200800273

[B26] Jones M Cordell JL Beyers AD Tse AG Mason DY Detection of T and B cells in many animal species using cross-reactive anti-peptide antibodies J Immunol 1993 150 5429 5435 8515069

[B27] Witte F Fischer J Nellesen J Vogt C Vogt J Donath T Beckmann F In vivo corrosion and corrosion protection of magnesium alloy LAE442 Acta Biomater 2010 6 1792 1799 10.1016/j.actbio.2009.10.012 19822226

[B28] Song G Atrens A Corrosion mechanisms of magnesium alloys Adv Eng Mater 1999 1 11 33 10.1002/(SICI)1527-2648(199909)1:1<11::AID-ADEM11>3.0.CO;2-N

[B29] Witte F Hort N Vogt C Cohen S Kainer KU Willumeit R Feyerabend F Degradable biomaterials based on magnesium corrosion Curr Opin Solid State Mater Sci 2008 12 63 72 10.1016/j.cossms.2009.04.001

[B30] Zhang S Zhang X Zhao C Li J Song Y Xie C Tao H Zhang Y He Y Jiang Y Bian Y Research on an Mg-Zn alloy as a degradable biomaterial Acta Biomater 2010 6 626 640 10.1016/j.actbio.2009.06.028 19545650

[B31] Edwards JC Sedgwick AD Willoughby DA The formation of a structure with the features of synovial lining by subcutaneous injection of air: an in vivo tissue culture system J Pathol 1981 134 147 156 10.1002/path.1711340205 7019400

[B32] Goldring SR Schiller AL Roelke M Rourke CM O'Neil DA Harris WH The synovial-like membrane at the bone-cement interface in loose total hip replacements and its proposed role in bone lysis J Bone Joint Surg Am 1983 65 575 584 6304106

[B33] Ko CY Ahn CY Ko J Chopra W Shaw WW Capsular synovial metaplasia as a common response to both textured and smooth implants Plast Reconstr Surg 1996 97 1427 1435 10.1097/00006534-199606000-00017 8643727

[B34] Werkmeister JA Tebb TA White JF Ramshaw JAM Collagenous tissue formation in association with medical implants Curr Opin Solid State Mater Sci 2001 5 185 191 10.1016/S1359-0286(01)00007-9

[B35] Ungersböck A Perren SM Pohler O Comparison of the tissue reaction to implants made of a beta titanium alloy and pure titanium. Experimental study on rabbits J Mater Sci Mater Med 1994 5 788 792 10.1007/BF00213136

[B36] Meachim G Williams DF Changes in nonosseous tissue adjacent to titanium implants J Biomed Mater Res 1973 7 555 572 10.1002/jbm.820070607 4589049

[B37] LeGeros RZ Craig RG Strategies to affect bone remodeling: osteointegration J Bone Miner Res 1993 8 Suppl 2 S583 S596 8122530 10.1002/jbmr.5650081328

[B38] Ungersböck A Pohler OE Perren SM Evaluation of soft tissue reactions at the interface of titanium limited contact dynamic compression plate implants with different surface treatments: an experimental sheep study Biomaterials 1996 17 797 806 10.1016/0142-9612(96)81417-7 8730964

[B39] Anderson JM Rodriguez A Chang DT Foreign body reaction to biomaterials Semin Immunol 2007 20 86 100 10.1016/j.smim.2007.11.004 18162407 PMC2327202

[B40] Anderson JM Chapter 4 - Mechanisms of inflammation and infection with implanted devices Cardiovasc Pathol 1993 2 33S 41S 10.1016/1054-8807(93)90045-4

[B41] Gogolewski S Bioresorbable polymers in trauma and bone surgery Injury 2000 31 28 32 10.1016/S0020-1383(00)80020-0 11270078

[B42] Böstman OM Intense granulomatous inflammatory lesions associated with absorbable internal fixation devices made of polyglycolide in ankle fractures Clin Orthop Relat Res 1992 278 193 199 1314146

[B43] Weiler A Hoffmann RF Stähelin AC Helling HJ Südkamp NP Biodegradable implants in sports medicine: the biological base Arthroscopy 2000 16 305 321 10.1016/S0749-8063(00)90055-0 10750011

[B44] Hoffmann R Krettek C Hetkämper A Haas N Tscherne H Osteosynthesis of distal radius fractures with biodegradable fracture rods. Results of two years follow-up Unfallchirurg 1992 95 99 105 1315080

[B45] Banchereau J Brière F Liu YJ Rousset F Molecular control of B lymphocyte growth and differentiation Stem Cells 1994 12 278 288 10.1002/stem.5530120304 7521239

[B46] Voggenreiter G Leiting S Brauer H Leiting P Majetschak M Bardenheuer M Obertacke U Immuno-inflammatory tissue reaction to stainless-steel and titanium plates used for internal fixation of long bones Biomaterials 2003 24 247 254 10.1016/S0142-9612(02)00312-5 12419625

